# Bleeding duodenal varices urgently treated with TIPS and coil embolization in a patient with cirrhosis: A rare case

**DOI:** 10.1016/j.radcr.2021.03.046

**Published:** 2021-04-21

**Authors:** Monica Palermo, Serafino Santonocito, Giovanni Failla, Francesco Vacirca, Massimo Venturini, Stefano Palmucci, Antonio Basile

**Affiliations:** a Radiology Unit 1, Department of Medical Surgical Sciences and Advanced Technologies "GF Ingrassia," University-Hospital Policlinico "G. Rodolico-S. Marco", University of Catania, Via S. Sofia, 95126, Catania, Italy; b Department of Diagnostic and Interventional Radiology, University of Insubria, Ospedale di Circolo e Fondazione Macchi, Varese, Italy

**Keywords:** Interventional radiology, Ectopic varices, Gastrointestinal bleeding, Endovascular treatment, TIPS, Coil embolization

## Abstract

Duodenal varices are ectopic varices that can cause severe and life-threatening gastrointestinal bleeding. Diagnosis and treatment of ectopic varices is challenging, because endoscopy is often unproductive in detecting and treating ectopic varices. Interventional radiology appears as an alternative in this setting, thanks to its important role in treating the bleeding caused by ectopic varices and in preventing rebleedings. We present an interesting case of bleeding ectopic varices in a male 62-years-old cirrhotic patient (Child-Pugh B8). The patient presented with hemorrhagic shock caused by massive melena. CT angiography showed intraluminal blood and identified the source of bleeding as an ectopic varicose vein draining into the superior mesenteric vein. Interventional radiology approach was the only applicable one to reach and effectively treat the source and cause of bleeding by TIPS placement and embolization of the collateral feeding at the same session

## Introduction

Duodenal varices are a rare cause of gastrointestinal bleeding, often caused by portal hypertension. Portal hypertension is a dangerous and common complication of liver cirrhosis which can lead to abnormal variceal collaterals between the portal and systemic circulation [1]. The most common sites of variceal formation are gastro-esophageal varices (esophageal and cardio-fundic varices) [2]; varices occurring in other sites are defined as ectopic varices. Ectopic varices are a relatively rare cause of bleeding (2%-5% of variceal bleeding); among ectopi varices, the duodenum is the most common site of bleeding, followed by the jejunum and ileum, colon, and rectum [1,3]. Ectopic varices bleeding represents a life-threatening condition, with a 4-fold increased bleeding risk than esophageal varices and a mortality rate up to 40% [2]; its diagnosis can be challenging and standard local therapy is not always achieved due to anatomical unattainability [4].

Treatment strategies include medical therapy, local ligation, sclerotherapy, surgery, or percutaneous treatment. The choice of the best option lies in different factors, like the site of bleeding, patient's condition and the cause of portal hypertension [1,^3^,5].

As stated by the recent Consensus Conference on TIPS management [6], percutaneous treatment options, such as coil embolization and transjugular intrahepatic porto-systemic shunt (TIPS), represent an alternative and have a potential role in the management of bleeding ectopic varices . TIPS can be considered as an efficacious option for ectopic varices bleeding management and for preventing rebleeding from ectopic varices, with the cost of being technically challenging and raising the risk of encephalopathy.

TIPS placement allows treating the underlying cause of bleeding (portal hypertension) and performing the embolization of the feeding vessel(s) with coils, which, therefore, may be performed as a combined procedure after TIPS placement [5]. However, it should be noted that ectopic varices are known for the high risk of rebleeding despite a reduction of HVPG below 12 mm Hg following TIPS [6].

We present a case of bleeding ectopic varices in a cirrhotic patient, treated with TIPS and embolization of the collateral feeding at the same session.

## Case Presentation

A 62-year-old man, with alcohol-related cirrhosis, presented with massive melena causing anemia (Hb 6.9 mg/dL), hemorrhagic shock, lethargic mental status and hemodynamic instability (HR 120, BP 90/60). His Child-Pugh class was B8.

**Fig. 1:**
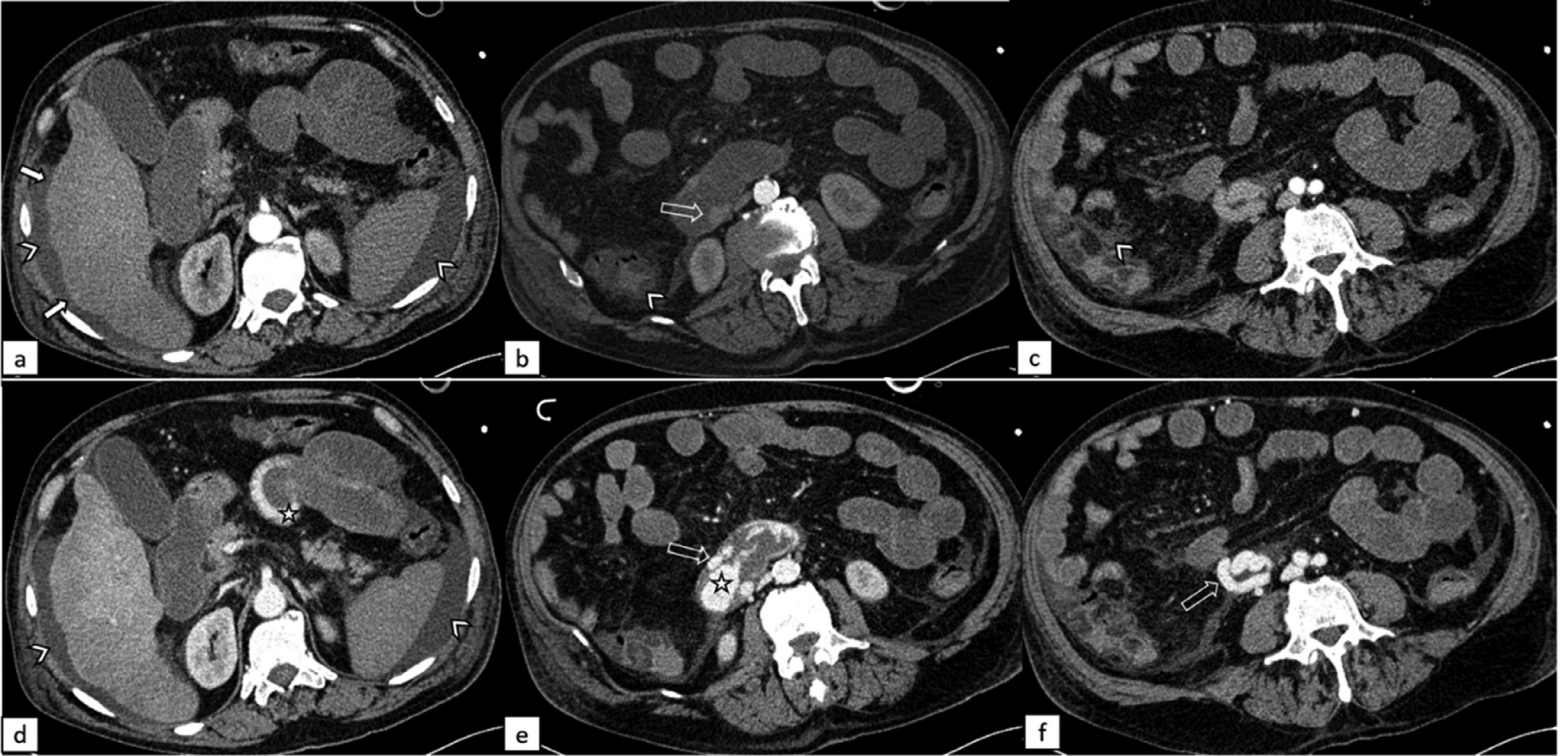
Contrast enhanced computed tomography of the abdomen. Same three slices in the arterial phase (above) and portal phase (below), showing signs of cirrhosis (arrowheads: ascites; white arrows: liver surface undulations), venous varices (empty arrows) in the third section of the duodenum (b, e, f) and intraluminal active bleeding (star).

**Fig. 2:**
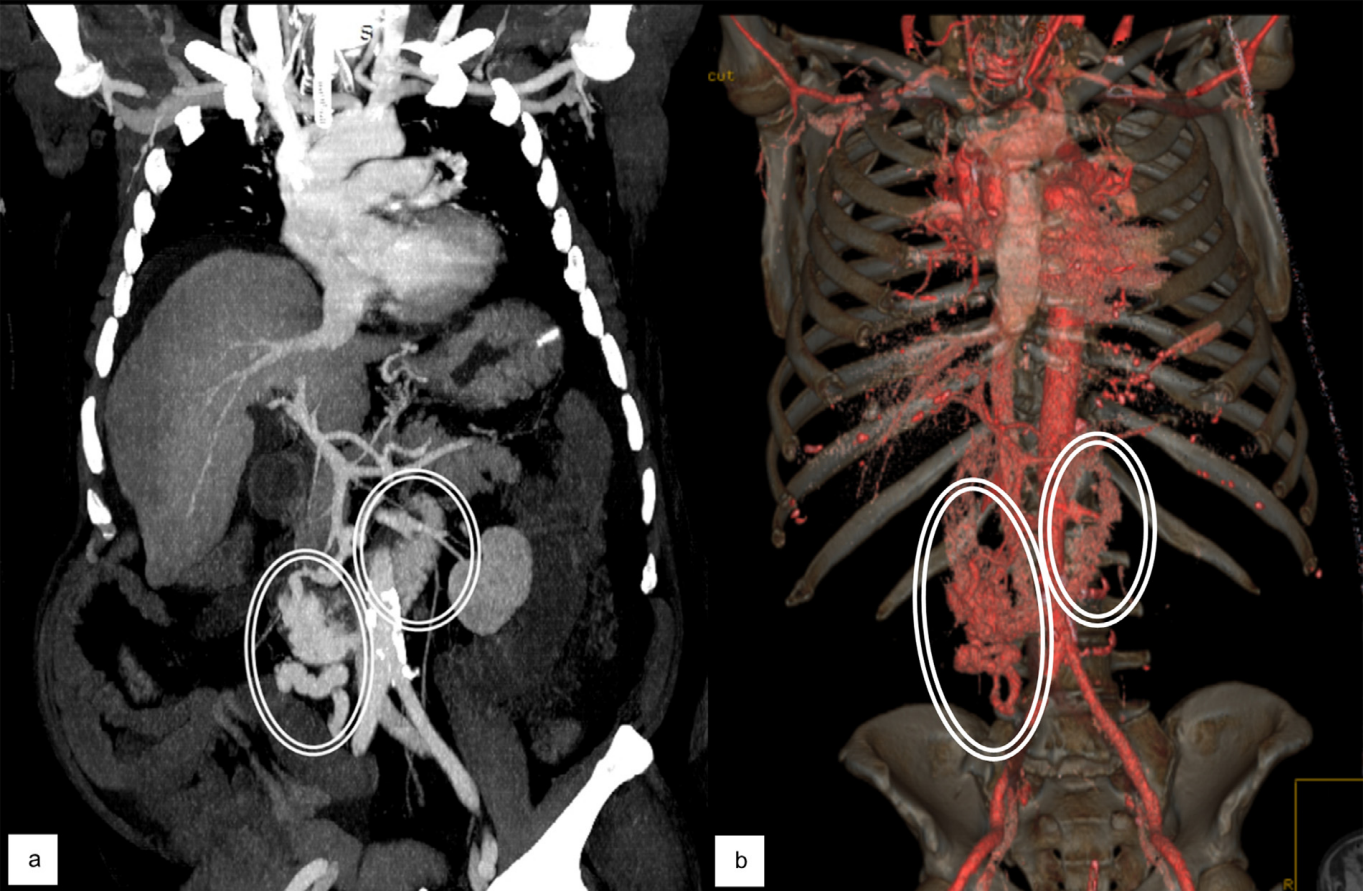
Coronal maximum intensity projection (MIP) (a) and 3d reconstruction (b) from contrast-enhanced CT demonstrating the presence of tortuous enlarged varicose vein with intraluminal duodenal and jejunal enhanced material due to active bleeding (circles).

**Fig. 3:**
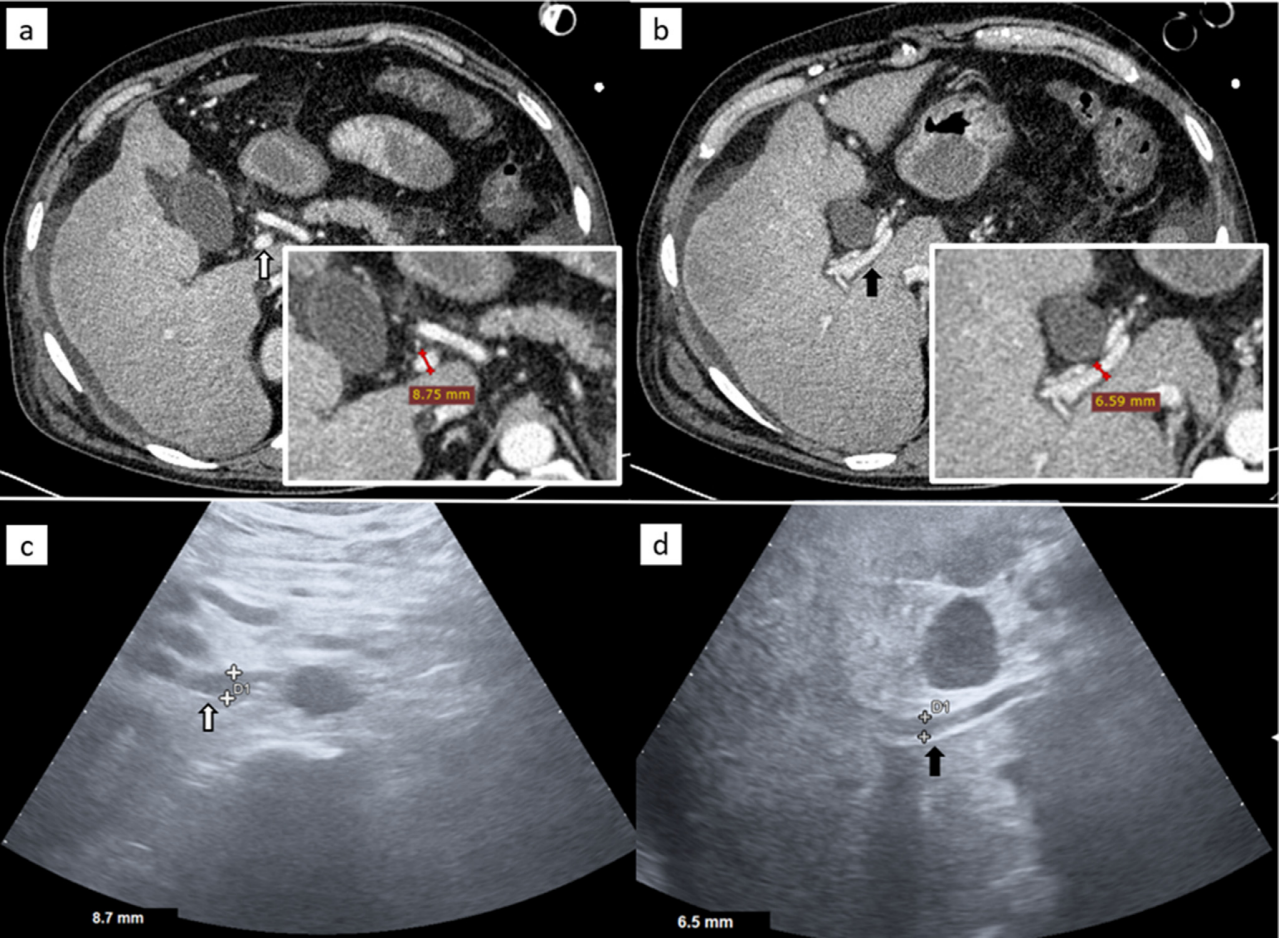
Contrast enhanced computed tomography (CECT) and corresponding ultrasound (US) imaging. White arrow: extrahepatic tract of the portal vein and its measurement in CECT (a) and in US (c). Black arrow: intrahepatic tract of s portal vein and its measurement in CECT (b) and in US (c). Red line: CT caliper; White + and D1: US caliper.

A CT angiography (CTA) was performed (Figs. 1, 2). It showed the presence of intraluminal blood, from the gastric fund to the rectum, and identified the source of bleeding as a contrast medium leakage at the level of the third section of the duodenum from a dilatated and tortuous varicose vein draining into the superior mesenteric vein. The cirrhotic liver was confirmed, the spleen was enlarged and there was transudative ascites. CT scan also proved diffuse vasoconstriction, due to shock status, with a very tiny portal vein (7 mm) (Fig. 3a and b).

An endoscopic attempt was performed, but the source of bleeding wasn't located.

As the patient was hemodynamically unstable, he was urgently transferred to the angiographic suite and, after resuscitation maneuvers, ultrasonography was performed: it showed a 6 mm portal vein (Fig. 3c and d).

**Fig. 4:**
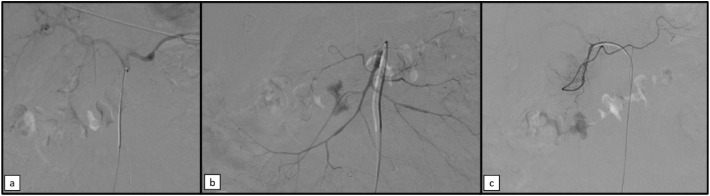
Selective arteriography of the celiac trunk (a), superior mesenteric artery (b) and gastroduodenal artery (c) showed no signs of arterial bleeding of arteriovenous malformations, but demonstrated signs of vasospasm due to hemorrhagic shock.

**Fig. 5:**
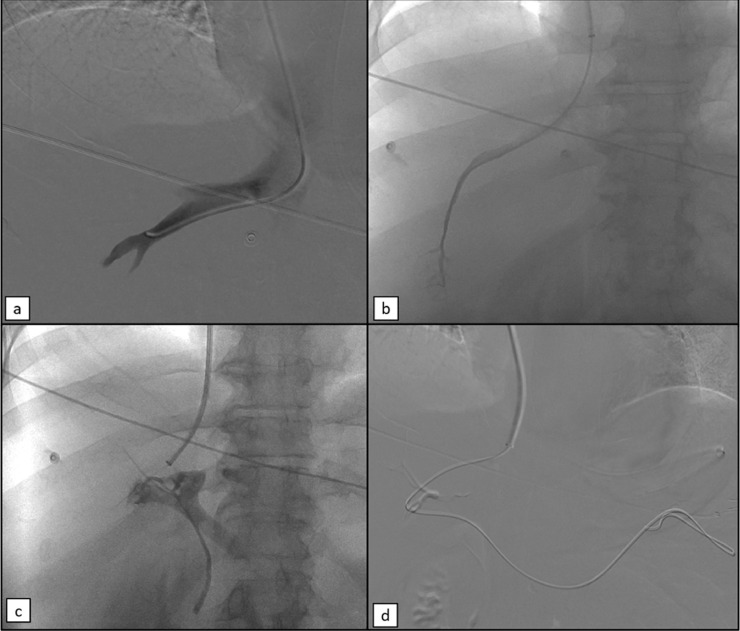
First steps of TIPS. (a) Phlebography of the middle hepatic vein. (b) Wedged catheter for the HVPG measurement. (c) Puncture of the portal vein using a 14-G needle: even the biliary tract was opacified in this frame. (d) 0.035” guidewire pushed into the splenic vein.

**Fig. 6:**
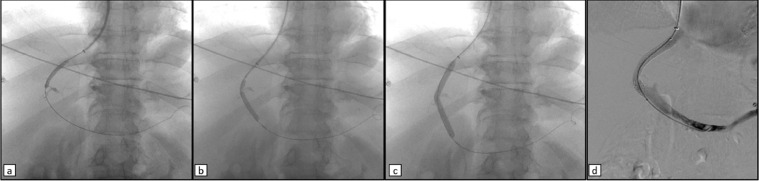
Last steps of TIPS. (a, b, c) Subsequent progressive balloon dilatations of the intra-epathic tract, allowing the passage of the 10 French sheath into the portal vein. (d) Final check after the deployment and the post-dilatation of the 8-10 mm × 7 + 2 cm PTFE-covered stent (Viatorr, W. L. Gore).

Initially, in the doubt of an arteriovenous malformation (AVM) as the cause of the massive bleeding, arteriography by right femoral access was performed (Fig 4). Selective angiography of the celiac and superior mesenteric artery was performed and no findings of AVM or arterial hemorrhage were noted. Subsequently, right jugular access was gained and a long sheath was placed, in order to navigate to the middle hepatic vein through the inferior cava vein (ICV). Then, hepatic venous pressure gradient (HVPG) was measured and found of 21 mm Hg. Therefore, an urgent TIPS was performed. The portal vein was reached and catheterized, and the intrahepatic tract was dilatated with an 8 mm balloon catheter. A Viatorr stent (8-10mm x 7+2 cm) was then placed. After the TIPS procedure, HVPG was 11 mmHg. As a consequence, the TIPS procedure was successfully performed. All the steps of the TIPS procedure are illustrated in Figs. 5 and 6.

**Fig. 7:**
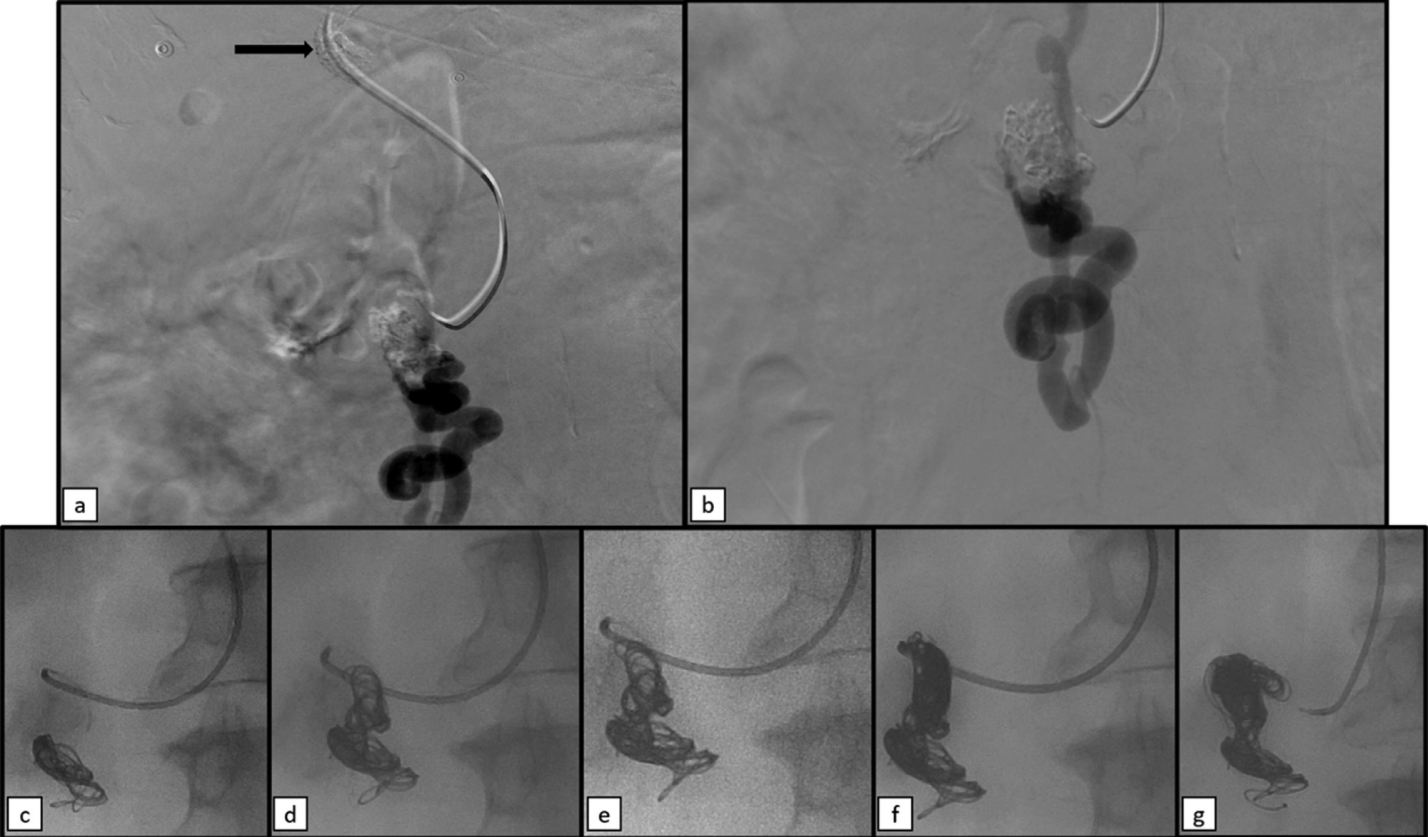
Selective venography of superior mesenteric vein showing the tortuous and enlarged vein at the level of duodenum, without signs of active bleeding at the moment (black arrow in a: the distal tip of TIPS). From (c) to (g) Embolization performed through a 2.7" Progreat Terumo microcatheter, by placing Ruby Coils of 16 mm, 18 mm, 28 mm (e) and 32 mm and Packing Coil Penumbra.

Afterward, the superior mesenteric vein was reached through the just gained access and a selective venogram of the enlarged vein was performed (Figure 7a and b). There was no evidence of active bleeding at the moment of angiography but, in consideration of the high risk of rebleeding of ectopic varices, embolization was performed with the placement of 16, 18, 28 e 32 mm Ruby Coil and Packing Coil Penumbra through a 2.7" Progreat Terumo microcatheter (Figure 7c-g).

The final technical result was impeccable.

The patient was then transferred to the Intensive Care Unit. Regrettably, the patient died after 3 days, for neurological and septic complications due to prolonged hemorrhagic shock.

## Discussion

Ectopic varices (may occur at various abdominal sites and their prevalence is higher in patients with portal hypertension, in particular for duodenal varices. Ectopic varices are a rare but serious cause of gastrointestinal bleeding and their diagnosis could be challenging, as endoscopy could be unproductive in detecting ectopic varices [7]. Treatment options include local endoscopic, radiologic or surgical procedures, and portal decompressive surgery or TIPS. As in our case, local endoscopic treatment is often impossible or ineffective [6]. Therefore, a radiological approach can and should be considered, as supported by literature evidence [4,[8], [9], [10], [11], [12].

Radiologic procedures allow treating the underlying cause of the bleeding by TIPS placement, thereby controlling the bleeding itself, by using embolizing agents. TIPS should be considered early to reduce hepatic venous pressure gradient, to achieve portal venous decompression and to improve variceal bleeding control [13].

Variceal coil embolization is a safe and technically feasible option to embolize the feeding vessel leading to the ectopic varices. As suggested by Macedo 2005 [5], it should be performed at the same time of TIPS placement.

In our case, TIPS combined with embolization has been an effective therapy for controlling bleeding from ectopic varices.

Physicians and radiologists should keep in mind this uncommon cause of life-threatening gastrointestinal bleeding. As endoscopy is not often suitable for ectopic varices, adequate management including interventional radiology should be taken into consideration to provide and achieve a prompt and proper treatment.
